# The Vienna idiopathic intracranial hypertension database—An Austrian registry

**DOI:** 10.1007/s00508-023-02252-x

**Published:** 2023-08-31

**Authors:** Philip Pruckner, Christoph Mitsch, Stefan Macher, Nik Krajnc, Wolfgang Marik, Klaus Novak, Christian Wöber, Berthold Pemp, Gabriel Bsteh

**Affiliations:** 1 Department of Neurology, Medical University of Vienna, Waehringer Guertel 18–20, 1090 Vienna, Austria; 2 Medical University of Vienna, Comprehensive Center for Clinical Neurosciences & Mental Health, Vienna, Austria; 3 Department of Ophthalmology, Medical University of Vienna, Vienna, Austria; 4 Department of Neuroradiology, Medical University of Vienna, Vienna, Austria; 5 Department of Neurosurgery, Medical University of Vienna, Vienna, Austria

**Keywords:** Pseudotumor cerebri, Database, Treatment, Outcome, Diagnostic findings

## Abstract

**Background:**

Idiopathic intracranial hypertension (IIH) is becoming increasingly more prevalent bearing the risk of visual impairment and affecting the quality of life. Clinical presentation and outcome are heterogeneous. Large, well-characterized cohorts are scarce.

**Objective:**

To characterize the clinical spectrum, diagnostic findings, therapeutic management, and outcome of IIH.

**Methods:**

We identified patients with IIH according to modified Friedman criteria treated at our center between 2014 and 2021. The Vienna IIH database is described in detail.

**Results:**

Of 113 patients 89% were female (mean age 32.3 years). Median body mass index (BMI) was 31.8, with 85% overweight (BMI > 25) and 5% were classified as IIH without papilledema. Headache was present in 84% and showed migraine features in 43%. Median opening pressure in lumbar puncture was 31 cmH_2_O. Pharmacotherapy (predominantly acetazolamide) was established in 99%, 56% required at least 1 therapeutic lumbar puncture and 13% a surgical intervention. After a median 3.7 years follow-up, 57% had achieved significant weight loss, papilledema was present in 59% and headache in 76% (58% improved). Comparing initial presentation to follow-up, perimetry was abnormal in 67% vs. 50% (8% worsened, 24% improved) and transorbital sonography in 87% vs. 65% with a median optic nerve sheath diameter of 5.4 mm vs. 4.9 mm. Median peripapillary retinal nerve fiber layer thickness decreased from 199 µm to 99 µm and ganglion cell layer volume from 1.13 mm^3^ to 1.05 mm^3^.

**Conclusion:**

The large representative Vienna IIH cohort characterizes IIH-related symptoms, diagnostic findings, treatment, and outcome emphasizing substantial long-term sequelae of IIH. Future analyses will aim to refine phenotyping and identify factors predicting outcome.

## Introduction

Idiopathic intracranial hypertension (IIH), formerly known as pseudotumor cerebri, is a syndrome of increased intracranial pressure (ICP) of unclear etiology mainly affecting overweight women of childbearing age [1]. Although IIH is often considered rare, the estimated prevalence ranges up to 76 per 100,000 people and the incidence is increasing, especially in areas with high rates of obesity [2, 3]. Patients with IIH usually present with symptoms and signs of increased ICP commonly comprising headache and transient visual obscuration, while signs typically include papilledema. Of note, up to 25% of patients are asymptomatic and papilledema is incidentally discovered during routine eye examination [4]. While different diagnostic criteria for IIH have been proposed, the modified Friedman criteria are most commonly used [5]. The main hazard of IIH is persistent visual impairment occurring in up to 40% with 1–10% developing irreversible blindness [6, 7]. IIH also affects the quality of life beyond visual function by various symptoms, most prominently chronic headache [8]. Main treatment goals in IIH are to preserve visual function and alleviate symptoms. Evidence-based treatment options involve weight loss and medicinal treatment with acetazolamide [9]. Surgical intervention, usually cerebrospinal fluid (CSF) shunting, is required for IIH patients at acute risk of persistent visual impairment but may also be needed in those who fail to improve or worsen despite maximum tolerated medical therapy [10]. Overall, most patients show a chronic course of disease, accompanied with a significant reduction of quality of life, often resulting in frequent consultations for healthcare, and considerable direct and indirect socioeconomic costs [11]. In this context, establishment of adequate, evidence-based diagnostic and therapeutic patient management is of paramount importance; however, reports on large and well characterized cohorts are scarce.

The aim of this work is to describe the structure and methods of a newly established database of IIH patients and characterize the included cohort.

## Methods

### Patients and definitions

In November 2021, the Vienna IIH (VIIH) database was established at the Departments of Neurology and Ophthalmology, Medical University of Vienna, which serve as both primary and reference centers mainly for Vienna and its geographical catchment area. By February 2023, a cohort of 113 patients diagnosed with IIH or IIH without papilledema (IIH-WOP) according to modified Friedman criteria was included [5].

The prevalence of IIH in Europe is around 1–2 per 100,000 people [12]. Given a population of nearly 3 million people in the greater Vienna area, this study is likely to have caught most IIH patients from this geographic area. Patients presented initially either at the Department of Neurology or at the Department of Ophthalmology and were referred obligatorily to the other department. Experienced neurologists and neuro-ophthalmologists performed all examinations.

Standardized VIIH case reports including demographic data, disease-specific parameters as well as documentation of diagnostic and therapeutic procedures were completed.

Data are collected retrospectively from clinical reports at first visit and whenever the patient returns for scheduled follow-up or unscheduled visits. All initial assessments and follow-ups after the official establishment of the VIIH database (30-NOV-2021) are collected prospectively.

Initial neurologic assessment included a detailed patient history focussed on IIH-related symptom. Disease-specific parameters analyzed in this study were headache, nausea, visual problems, auditory problems, medication as well as risk factors and other symptoms. We measured symptom frequency in days per month or semiquantitatively using the following categories: “once”, “rarely” (< 7 days per month), “sometimes” (8–15 days per month), “frequently” (≥ 15 days per month), and “constantly”. Symptom intensity was rated between 1–10 based on the numeric rating scale (NRS) or the most appropriate semiquantitative category of “mild” (NRS 1–3), “moderate” (NRS 4–6), and “severe” (NRS 7–10). For symptom courses, we distinguished between “attack-like”, “undulating,” “constant,” and “progressive.” To characterize headaches, we distinguished between pressing, pulsating, or stabbing as well as unilateral and bilateral headaches. Furthermore, we used the collected information to categorize headache phenotypes according to the 3rd edition of the International Classification of Headache Disorders (ICHD-3) into migraine, tension type headache and other headaches [4]. For visual disturbances, we specified character, duration, course, frequency, laterality, and occurrence of obscuration. Characterizing visual disturbances, black vision, blurred vision, or double vision were distinguished as well as unilateral and bilateral visual problems.

### Ophthalmological assessment

Ophthalmological assessment obligatorily comprised visual acuity, fundoscopy, perimetry, and optical coherence tomography (OCT) with optional ocular ultrasonography. Best-corrected visual acuity was assessed using Sloan charts at distance after subjective refraction. Results are given in the logarithm of the minimum angle of resolution (logMAR). Meaningful change was defined as ≥ 0.2 logMAR [13].

Perimetry was performed by automated Humphrey visual field testing using the 30‑2 Swedish Interactive Threshold Algorithm (SITA), quantifying mean deviation in decibels (dB) compared to age-matched controls and defining abnormal perimetry as a mean deviation lower than −2 dB. Fundoscopy included assessment of papilledema and secondary optic atrophy. The Frisén staging scale was used to rate papilledema severity from stage 0 (no papilledema) to stage 5 (severe papilledema) [14].

The OCT imaging was done without pupil dilatation in a dark room on both eyes of each patient using the same spectral domain OCT (Heidelberg Engineering, Heidelberg, Germany; software Heidelberg eye explorer software version 6.9a) adhering to the OSCAR-IB quality control criteria [15]. Peripapillary retinal nerve fiber layer (pRNFL) thickness was measured using a standard 12° (3.4 mm) centered on the optic nerve head comprised 1536 A-scans with 100 averaged frames in automatic real-time tracking (ART). Ganglion cell layer volume was determined by a 20° × 20° macular volume scan centered on the macula and based on 512 A-scans and 25 B-scans aligned vertically with 16 averaged frames in ART in the 4 inner and outer quadrants of the circular grid. Image processing was semiautomated using the built-in proprietary software for automated layer segmentation and manual correction of obvious errors.

For assessment of the optic nerve sheath diameter (ONSD), we performed transbulbar sonography (Cinescan S, Quantel Medical, Clermont-Ferrand, France). First, we used a B-scan with the 10 MHz probe placed temporally to visualize the optic nerve transversely and radially in the coronal plane. A clearly differentiable echo-poor image of the optic nerve section taking on the aspect of a bat due to widened acoustic shadows is called a bat sign indicating perineural CSF congestion. Quantitative measurement of ONSD was done using standardized amplitude modulation (A-scan) echography with tissue sensitivity settings, placing the 8 MHz A-scan probe on the temporal eye equator in primary gaze position [16]. At least two measurements were taken within 3 mm of the posterior bulb wall, and the highest was documented as the diameter.

### Magnetic resonance imaging

Magnetic resonance imaging (MRI) was done using T1 or T2w sequences for excluding structural lesions and magnetic resonance angiography for excluding suspected sinus vein thrombosis or sinus vein stenosis. We recorded findings of an empty sella sign, decreased pituitary gland height, increased optic disc diameter, elongations of the optic nerve, flattening of the posterior sclera or sinus vein abnormalities.

### Lumbar puncture

At first admittance, we routinely performed diagnostic lumbar puncture. CSF parameters obtained were total cell count, albumin quotient and intrathecal synthesis of immunoglobulins. CSF opening pressure was measured with the patient in a supine right-sided position with legs extended. Abnormal CSF opening pressure was defined as values of > 250 mm CSF [5].

### Treatment

Therapeutic strategies comprised weight loss as well as pharmacological treatment. If performed, therapeutic CSF punctures and surgical interventions were documented.

### Follow-up

Standardized VIIH follow-up case reports use the same parameters as for diagnosis including neurological outcomes and ophthalmological assessments at every visit. For this study, we used the last respective documentation as last follow-up.

### Ethics

The study was approved by the ethics committee of the Medical University Vienna (approval number: 2216/2020). As this is a retrospective documentation of clinical routine data, the need for written informed consent from study participants was waived by the ethics committee.

### Data availability statement

Data supporting the findings of this study are available from the corresponding author upon reasonable request by a qualified researcher and upon approval by data-clearing unit of the Medical University Vienna.

### Statistics

For this study, we analyzed parameters during the period between initial visit, diagnosis and last documented follow-up applying an intention-to-treat approach. To mitigate potential biases of partly analyzing retrospective clinical data, we thoroughly applied quality control. Specifically, collected data were inspected for outliers by two independent investigators. Furthermore, we re-evaluated a random sample of 10% of enrolled patients to confirm the quality of initial data acquisition. Statistical analysis was performed using SPSS 26.0 (SPSS Inc, Chicago, IL, USA). Descriptive analyses were applied expressing categorical variables in absolute frequencies and percentages, continuous parametric variables as mean and standard deviation (SD) and continuous non-parametric variables as median with interquartile range (IQR) and absolute range (AR) as appropriate.

## Results

### Cohort characteristics

Applying modified Friedman criteria, the final VIIH cohort included 113 patients, of which 101 (89.4%) were female. Of those, 67 (59.2%) received a diagnosis before the cut-off date (30 November 2021) and were therefore included retrospectively, while 46 (40.7%) were included prospectively. Mean age at diagnosis was 32.3 years (SD 10.7) and median BMI 31.8 (IQR 27.6–39.9). Detailed characteristics of the study cohort are given in Table 1.

**Table 1:** Characteristics of 113 patients with IIH

		(*n* = 113)
**Females**	*n* (%)	101 (89.4)
**Age at diagnosis (years)**	Mean (SD)	32.3 (10.7)
**IIH with papilledema**	*n* (%)	107 (94.7)
**IIH without papilledema**	*n* (%)	6 (5.3)
**Symptoms at initial presentation**		
*Headache*	*n* (%)	95 (84.1)
Features of migraine	*n* (%)	41 (43.2)
Features of tension-type headache	*n* (%)	16 (16.8)
Unspecified	*n* (%)	38 (40.0)
*Visual symptoms*	*n* (%)	86 (76.1)
*Abducent nerve palsy*	*n* (%)	6 (5.3)
*Divergence insufficiency*	*n* (%)	4 (3.6)
*Pulsatile tinnitus*	*n* (%)	27 (23.9)
**BMI**	Median (IQR; AR)	31.8 (27.6–39.8; 17.3–60.9)
Overweight (BMI > 25)	*n* (%)	96 (85.0)
Obesity (BMI > 30)	*n* (%)	60 (53.1)
**Other risk factors and comorbidities**		
Hormonal contraception	*n* (%)	9 (8.0)
Tetracyclines	*n* (%)	2 (1.8)
Retinoids	*n* (%)	1 (0.9)
Hypothyroidism	*n* (%)	21 (18.6)
Diabetes mellitus	*n* (%)	8 (7.1)
Arterial hypertension	*n* (%)	14 (12.4)
COPD	*n* (%)	1 (0.9)
OSAS	*n* (%)	3 (2.7)
Depression	*n* (%)	19 (16.8)

*AR* absolute range, *IQR* interquartile range, *SD* standard deviation, *BMI* body mass index, *COPD* chronic obstructive pulmonary disorder, *IIH* idiopathic intracranial hypertension, *OSAS* obstructive sleep apnea syndrome

### MRI findings at initial diagnosis

Reports on MRI were available for 108 patients at initial diagnosis. The most commonly reported findings were an empty sella sign in 39.8%, transverse sinus stenosis in 29.2%, optic nerve sheath distension in 27.4%, optic nerve vertical tortuosity in 11.5% and posterior globe flattening in 3.5%.

### Neuro-ophthalmological findings at initial diagnosis

The vast majority of patients had papilledema at initial presentation (89%), with a median Frisén grade of 3 (IQR 2–4). Perimetry was abnormal in 66.7%, with a median deviation of −3.1 dB (IQR −11.6–−1.1); however, visual acuity was preserved in most patients (84%). Detailed neuro-ophthalmological findings including baseline parameters for OCT and ultrasonography are given in Table 2.

**Table 2:** Neuro-ophthalmological findings at initial diagnosis in 113 patients with IIH

*Visual acuity*	**–**	*n* = 106
Abnormal	*n* (%)	17 (16.0)
Worse eye (logMAR)	Median (IQR; AR)	0 (−0.1–0.0; −0.1–1.0)
*Perimetry*	**–**	*n* = 96
Abnormal	*n* (%)	64 (66.7)
Mean deviation in perimetry of worse eye (dB)	Median (IQR; AR)	−3.1 (−11.6–−1.1; −32.6–0)
*Fundoscopy*	**–**	*n* = 103
Abnormal	*n* (%)	98 (89.1)
Frisén scale	Median (IQR; AR)	3 (2–4; 0–5)
*Optical coherence tomography*	**–**	*n* = 99
pRNFL thickness (µm)	Median (IQR; AR)	199 (132–299; 55–560)
GCL volume (mm^3^)	Median (IQR; AR)	1.13 (1.05–1.17; 0.60–1.25)
*Ultrasonography*	**–**	*n* = 58
ONSD > 4.5 mm and/or bat sign	*n* (%)	53 (86.9)
ONSD of worse eye (mm)	Median (IQR; AR)	5.4 (5.0–5.7; 3.9–7.3)
ONSD intereye difference (mm)	Median (IQR; AR)	0.15 (0.04–0.28; 0.00–0.82)

*AR* absolute range, *IQR* interquartile range, *dB* decibels, *GCL* ganglion cell layer, *logMAR* logarithm of the minimum angle of resolution, *ONSD* optic nerve sheath diameter, *pRNFL* peripapillary retinal nerve fiber layer.

### Treatment

Significant weight loss (≥ 6%) was achieved in 57.0% with a median reduction of 7 kg (IQR 1–15), which equals a median 6.8% weight loss (IQR 1.3–14.9). Medicinal treatment was established in all 113 patients, of whom 99.1% received acetazolamide at median maximum dosage of 750 mg (IQR 500–1000). Overall, 55.8% received at least 1 therapeutic lumbar puncture after the initial diagnostic LP and 26.2% of these punctures were performed > 4 weeks after diagnosis. Surgical intervention was performed in 15 patients (13.3%), 14 of whom received a ventricular peritoneal/atrial shunt. Detailed treatment descriptions are given in Table 3.

**Table 3:** Weight loss, pharmacotherapy and surgical treatment in 113 patients with IIH

**Weight loss**	*n*	86
*Weight loss ≥* *6% from weight at diagnosis*	*n* (%)	49 (57.0)
*Change of weight absolute (kg)*	Median (IQR; AR)	−7 (−15–−1; −75–43)
*Change of weight percentage (%)*	Median (IQR; AR)	−6.8 (−14.9–−1.3; −58.6–40.2)
**IIH specific medication**	*n*	113
*Acetazolamide*	*n* (%)	112 (99.1)
Maximum acetazolamide dosage (mg)	Median (IQR; AR)	750 (500–1000; 250–2000)
*Topiramate*	*n* (%)	15 (13.3)
Maximum topiramate dosage (mg)	Median (IQR; AR)	62.5 (50–75; 25–200)
*Furosemide*	*n* (%)	12 (10.6)
Maximum furosemide dosage (mg)	Median (IQR; AR)	35.0 (20–40; 20–160)
*Combination treatment*	*n* (%)	23 (20.4)
**Therapeutic lumbar puncture**	*n*	113
*Entire study period*^a^	*n* (%)	63 (55.8)
Cumulative number^a^	Median (IQR; AR)	1 (0–4; 0–26)
*Follow-up (>* *4 weeks after diagnosis)*	*n* (%)	27 (26.2)
Cumulative number	Median (IQR; AR)	0 (0–1; 0–23)
**Surgery**	*n*	113
*Ventricular peritoneal/atrial shunt*	*n* (%)	14 (12.4)
Shunt revision	*n* (%)	3 (21.4)
*Bariatric surgery*	*n* (%)	1 (0.9)

*IQR* interquartile range, *AR* absolute range

^a^not including initial diagnostic lumbar puncture

### Outcome

**Fig. 1:**
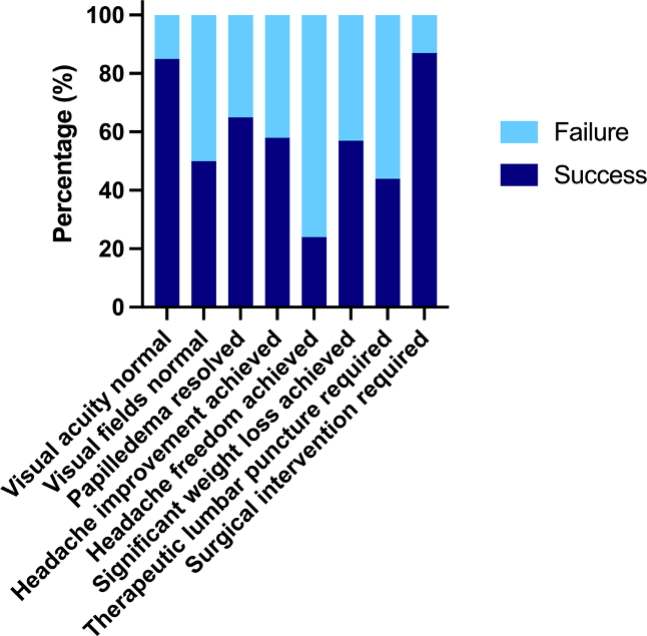
Overview outcome

Median duration of follow-up in our cohort was 3.7 years. At last follow-up, 76.0% still had headaches; however, 24.0% were headache-free and 57.7% reported a significant improvement of the headache. Visual acuity was abnormal in 15.0% and abnormal visual fields were documented in 49.6% (see Fig. 1).

In OCT, median pRNFL thickness was 99 µm (IQR 87–110) and median GCL volume was 1.05 mm^3^ (IQR 0.94–1.10), corresponding to a median change of −0.05 mm^3^. Ultrasonography follow-up showed an ONSD > 4.5 mm and/or a bat sign in 64.5% of patients. Detailed outcome data are given in Table 4.

**Table 4:** Diagram showing percentage of clinical and paraclinical outcome achieved in the study cohort

**Duration of follow-up (years)**	Median (IQR; AR)	3.7 (1.8–6.1; 0.3–17.4)
**Headache**	*n*	104
*Headache-free*	*n* (%)	25 (24.0)
*Persistent headache*	*n* (%)	79 (76.0)
*Improved*^a^	*n* (%)	60 (75.9)
**Visual acuity**	*n*	110
*Abnormal*	*n* (%)	17 (15.0)
*Worse eye (logMAR)*	Median (IQR; AR)	−0.1 (−0.1–0.0; −0.1–2.0)
*Change of visual acuity in worse eye (logMAR)*^a^	Median (IQR; AR)	0.0 (−0.1–0.0, −0.4–1.0)
Worsened^a^ (> +0.2 logMAR)	*n* (%)	5 (4.5)
Improved^a^ (> −0.2 logMAR	*n* (%)	5 (4.5)
**Perimetry (*n*** **=** **106)**		
*Abnormal*	*n* (%)	57 (49.6)
*Mean deviation of worse eye (dB)*	Median (IQR; AR)	−0.8 (−5.2–−0.3; −33.9–1.3)
*Change of mean deviation of worse eye (dB)*^a^	Median (IQR; AR)	1.6 (0.1–3.6; −10.8–25.4)
Worsened (> −2 dB)^a^	*n* (%)	8 (8.1)
Improved (> +2 dB)^a^	*n* (%)	27 (23.9)
**Fundoscopy (*n*** **=** **109)**		
*Abnormal*	*n* (%)	64 (59.3)
Worsened^a^	*n* (%)	5 (4.6)
Improved^a^	*n* (%)	62 (57.4)
**Optical coherence tomography (*n*** **=** **95)**		
*pRNFL thickness (µm)*	Median (IQR; AR)	99 (87–110; 29–245)
*Change of pRNFL (µm)*^a^	Median (IQR; AR)	−105 (−202–−30; −504–21)
*GCL volume (mm*^*3*^*)*	Median (IQR; AR)	1.05 (0.94–1.10; 0.62–1.13)
*Change of GCL volume (mm*^*3*^*)*^a^	Median (IQR; AR)	−0.05 (−0.14–−0.03; −0.52–0.09)
**Ultrasonography (*n*** **=** **76)**		
*ONSD >* *4.5* *mm and/or bat sign*	*n* (%)	49 (64.5)
Normalized ONSD (*n* = 56)	*n* (%)	7 (11.5)
*ONSD of worse eye (mm)*	Median (IQR; AR)	4.9 (4.3–5.3; 3.0–6.3)

*AR* absolute range, *dB* decibels, *GCL* ganglion cell layer, *IQR* interquartile range, *logMAR* logarithm of the minimum angle of resolution, *ONSD* optic nerve sheath diameter, *pRNFL* peripapillary retinal nerve fiber layer

^a^compared to baseline

## Discussion

This study aimed to describe the structure and methods of the VIIH database and to characterize a population-based real-world IIH cohort, which is the largest European IIH cohort presented to date.

The main findings align well with previously published literature: IIH is a rare disease, mainly affecting young overweight women. Most common symptoms at first admittance are headaches resembling migraine as well as visual disturbances. A multimodal treatment concept of weight loss and pharmacological treatment can improve disease course, but long-term sequelae of IIH may be substantial.

Acknowledging the limitations of retrospective data collected before the official establishment of the VIIH database, this study provides a comprehensive overview of demographics, clinical presentations, and disease courses of IIH in middle Europe.

### Cohort characteristics and symptoms

Demographic characteristics of the VIIH cohort are in line with previously published cohorts [17]. At initial presentation headache was present in 84% and resembling migraine in 43%. Reported rates of headache types vary in the literature. While Friedman et al. initially described that about 20% suffer from migraine-like headaches, the IIH treatment trial (IIHTT) cohort showed a prevalence of 52% migraine-like headaches [8, 18].

Visual symptoms were present in 76% at initial presentation and 95% had papilledema, while 5% were classified as IIH-WOP. Previous reports have also indicated a 5% proportion of patients with IIH without papilledema [19].

### Paraclinical findings

The MRI findings most frequently reported in our cohort were an empty sella sign (40%), transverse sinus stenosis (29%), and optic nerve sheath distension (27%). These frequencies are lower than in a recent study by Brodsky et al., which can likely be explained by the retrospective design of our study, where MRI reports are not standardized and depend on the indications for MRI, especially whether radiologists are specifically asked for signs of IIH [20].

Lumbar puncture opening pressure in our cohort (median 31 cmH_2_O) aligns well with findings from other studies [21, 22]. Here, it is important to stress that establishing a definite diagnosis of IIH requires measuring CSF opening pressure [5]. While this may be technically difficult in some cases (*n* = 8, 7.1% in our cohort) due to e.g., extensive subcutaneous tissue, CT-guided LP enabled measurement in all these cases. Proportions of abnormalities in visual acuity and visual fields (16% and 67%, respectively) are also similar to previous studies with ranges of 10–25% and 61–92%, respectively [23, 24]. As well, OCT findings (median pRNFL thickness 199 µm, median GCL volume 1.13 mm^3^) in our cohort are comparable to reported findings from other groups [25, 26]. Also, ultrasonography showed similar ONSD (median 5.4 mm) compared to previously published studies using standardized A‑scan echography in IIH, but slightly lower values than studies measuring ONSD from B‑scans (approximately 6.5 mm) [27, 28]. B‑scan ultrasound has limited resolution that depends on the frequency used, and as standardized settings are not available, caliper measurements in the images are influenced by the applied signal gain due to a blooming effect. Standardized A‑scan echography avoids these drawbacks and makes it a more accurate measure of ONSD.

### Treatment

In the VIIH cohort, significant weight loss was achieved in 57% of patients with a median reduction of about 7% from weight at initial presentation, while 43% did not achieve significant weight loss. Patients are routinely referred to endocrinology and dietology for counselling regarding weight management. In more severe or refractory cases, bariatric surgery is recommended [29].

All but one patient received acetazolamide treatment. In our practice, acetazolamide dosage is chosen based on a treat-to-target approach aiming to resolve papilledema. When this target is reached, acetazolamide is then gradually and slowly reduced under close monitoring while continuing weight management.

Therapeutic lumbar punctures were necessary in 56% and surgical treatment (mostly ventricular peritoneal/atrial shunt) in 13%. Achieved weight loss, average maximum acetazolamide dosage and proportion of lumbar punctures were largely in line with the therapeutic strategy of the acetazolamide plus weight loss arm of IIHTT [22]; however, additional lumbar punctures were performed electively after 6 months in a selected subpopulation and use of other medications, such as topiramate was prohibited [22].

### Outcome

Long-term follow-up after almost 4 years showed persistent headache in 76%, although headache had also improved in 76%. This is again in line with the IIHTT where headache remained present in 68% [22]. The proportion of patients with abnormal visual acuity remained at 15% compared to baseline, which underlines that central vision function is largely unaffected by moderately increased ICP [9]. Conversely, perimetry was abnormal in 67% at baseline compared to 50% at last follow-up (8% worsened, 24% improved) with a slight overall improvement (+2.1 dB) from baseline. Again, this is comparable to the improvement of +1.4 dB found in the IIHTT acetazolamide + weight loss group [22]. In OCT, pRNFL thickness was significantly reduced by a median 105 µm resulting in apparently normal pRNFL thickness at last follow-up (99 µm). While pRNFL cannot distinguish between real resolution of papilledema and pseudonormalization caused by atrophy, the corresponding finding of very moderate loss of GCL volume (−0.05 mm^3^ over nearly 4 years) strongly indicates a small degree of neuroaxonal damage. These results confirm the significant effect of weight loss and acetazolamide as described in the IIHTT OCT substudy [28]. In ultrasonography, 65% still displayed an ONSD above 4.5 mm and/or a bat sign compared to 87% at baseline. Median ONSD improved from 5.4 to 4.9 mm, which is comparable to other studies that reported an average decrease of 0.2–0.7 mm after 6 months of treatment [27, 28, 30].

### Strengths and limitations

The strengths of the VIIH database are the large number of patients and the close-meshed standardized follow-up over a long-term period. Duration of follow-up close to 4 years, which is significantly longer than in prospective trials such as the IIHTT, enables characterization of long-term sequelae. Also, our cohort comprises the whole clinical spectrum of IIH, whereas e.g., the IIHTT only included patients with mild visual loss and patients were excluded if progression of visual symptoms was observed [22].

However, there are some limitations. The retrospective analyses of data collected in clinical routine creates a variety of possible biases, although these are mitigated by the standardized data collection and thorough quality control applied within the VIIH. Considering the IIH prevalence in the geographic area, VIIH seems to have caught most of the IIH patients. Still, a potential selection bias towards patients with more benign courses and/or severely disabled patients, who both tend to stop attending clinics, cannot be completely excluded. Although acquired in a real-world cohort, OCT and echography scans were meticulously controlled for quality and confounding factors were rigorously ruled out, e.g., severe myopia, optic disc drusen or previous diagnoses of ophthalmological, neurological, systemic or drug-related causes of vision loss or retinal damage not attributable to IIH. Biological variability and measurement errors were also minimized by a homogeneous single center data set. These sources of error might be increased when protocols and devices vary, and multicenter data sets are used.

## Conclusion

The population based real-world VIIH cohort, the largest European IIH cohort presented to date, aligns well with findings previously published in the literature. The findings of this study reflect a representative overview of IIH-related symptoms, diagnostic findings, treatment, and outcome parameters and emphasize substantial long-term sequelae. Future analyses will aim to refine phenotyping of IIH patients and identify factors predicting outcome.
